# Rhizosphere bacterial and fungal communities during the growth of *Angelica sinensis* seedlings cultivated in an Alpine uncultivated meadow soil

**DOI:** 10.7717/peerj.8541

**Published:** 2020-03-26

**Authors:** Zhigang An, Fengxia Guo, Yuan Chen, Gang Bai, Zhengjun Chen

**Affiliations:** 1 College of Life Science and Technology, College of Agronomy, Gansu Provincial Key Lab of Good Agricultural Production for Traditional Chinese Medicine, Gansu Provincial Engineering Research Centre for Medical Plant Cultivation and Breeding, Provincial Key Lab of Aridland Crop Science, Gansu Agricultural University, Lanzhou, China; 2 Pharmacy Department, Gansu University of Chinese Medicine, Dingxi, China; 3 Gansu Engineering Lab of Resource Reservation and Utilization for Characteristic Chinese Medicine, Gansu Tasly Zhongtian Pharmaceutical Co., Ltd., Dingxi, China

**Keywords:** *Angelica sinensis*, Seedlings, Rhizosphere, Bacteria, Fungi, Diversity, Community function

## Abstract

**Background:**

*Angelica sinensis* seedlings are grown in alpine uncultivated meadow soil with rainfed agroecosystems to ensure the quality of *A. sinensis* after seedling transplantation. The aim was to investigate the rhizosphere bacterial and fungal communities during the growth stages of *A. sinensis* seedlings.

**Methods:**

The bacterial and fungal communities were investigated by HiSeq sequencing of 16S and 18S rDNA, respectively.

**Results:**

Proteobacteria and Bacteroidetes were bacterial dominant phyla throughout growth stages. Fungal dominant phyla varied with growth stages, dominant phyla Ascomycota and Chytridiomycota in AM5, dominant phyla Basidiomycota, Ascomycota and Zygomycota in BM5, and dominant phyla Basidiomycota and Ascomycota in CM5. There was no significant variation in the alpha-diversity of the bacterial and fungal communities, but significant variation was in the beta-diversity. We found that the variation of microbial community composition was accompanied by the changes in community function. The relative abundance of fungal pathogens increased with plant growth. We also identified the core microbes, significant-changing microbes, stage-specific microbes, and host-specific microbes. Plant weight, root length, root diameter, soil pH, rainfall, and climate temperature were the key divers to microbial community composition.

**Conclusions:**

Our findings reported the variation and environmental drivers of rhizosphere bacterial and fungal communities during the growth of *A. sinensis* seedlings, which enhance the understanding of the rhizosphere microbial community in this habitat.

## Introduction

*Angelica sinensis* (Oliv.), Diels (Umbelliferae), is an herbaceous perennial plant, widely used in natural medicines in China. In cultivation, it has a three-year growth cycle, fostering the seedlings in the first year, transplanting the seedlings and harvesting the fleshy roots in the second year, and collecting the seeds in the third year. Dingxi is the major producing area for *A. sinensis* in China, accounting for 70% of the country’s production each year.

Rhizosphere microbes are closely related to plant growth. They are considered the second genome of the plant and have pivotal functions in plant health and productivity, such as in nutrient cycling, pathogen suppression, growth promotion, and abiotic stress tolerance (Berendsen, Pieterse & Bakker, 2012; Strecker et al., 2016). Yet, the formation of rhizosphere microbe communities is affected by environmental factors such as soil pH (Hardoim et al., 2011), soil temperature (Lareen, Burton & Schafer, 2016) and plant development (Chaparro, Badri & Vivanco, 2014).

Much research has focused on rhizosphere microbial communities during the plant development, including microbial composition, community diversity, and core microbes (Tkacz et al., 2015; Vimal et al., 2017). Generally, the microbial communities around the roots of different plants are dominated by different microbial phyla, for example, the bacterial phyla Acidobacteria and Proteobacteria for black peppers (Xiong et al., 2015), and the bacterial phyla Proteobacteria, Actinobacteria and Acidobacteria as well as the fungal phyla Ascomycota, Zygomycota and Basidiomycota for apples (Franke-Whittle et al., 2015). Additionally, the alpha- and beta-diversities of the rhizosphere bacteria of potato plants are influenced differently by plant growth stage (Pfeiffer et al., 2017). In the rhizosphere fungal community of potato plants, alpha-diversity is stable but beta-diversity differs with growth stage (Zimudzi et al., 2018). Moreover, the core rhizosphere bacteria have been identified in potatoes (Pfeiffer et al., 2017), blueberries (Jiang et al., 2017), and *Arabidopsis* (Schlaeppi et al., 2014).

Rhizosphere communities are functionally diverse, which may be closely related to community composition (Zimudzi et al., 2018). Many microbes usually inhabit the rhizospheres of different plants and play a role in ecological functions, such as C, N and S cycling (Li et al., 2014; Fierer et al., 2012), indole acetic acid production (Kuffner et al., 2010), and biocontrol against plant-pathogenic fungi (Adrangi et al., 2010). However, many other microbes comprising bacteria and fungi are plant pathogens and they are not conducive to plant health (Peix, Ramirez-Bahena & Velazquez, 2018; McGovern et al., 2006).

Traditionally, *A. sinensis* seedlings are grown in alpine uncultivated meadow soil with rainfed agroecosystems to ensure seedling quality. Many studies have focused on the rhizosphere microbial communities of different plants, but currently, little is known about the rhizosphere microbial communities of *A.sinensis* seedling cultivated in this habitat. Thus, this study focused on bacterial and fungal communities during the growth stages and had three objectives: (1) to investigate global microbial diversity and potential microbial functions, (2) to find core microbes, significant-changing microbes, and specific microbes, and (3) to identify environmental factors driving the microbial community variation.

## Materials and Methods

### Study site and sample preparation

Seedling samples in the first year were collected from a study site near Dingxi (N 34°25′27″, E 104°28′24″, elevation 2,783 m). The study site is mountainous with a meadow soil and a rainfed agroecosystem. It has a cool and semi-humid climate with an annual average temperature of 5–6 °C, approximately 2,219 h annual sunshine, 90–120 frost-free days per year, and an annual rainfall of 451.4–817.8 mm which falls mainly from June to September.

In 2016 from June to October, the experiment was carried out. Rhizosphere samples were collected during the plant growth stage 56 days (AM5), 98 days (BM5), and 129 days (CM5) after planting. Three samples for each tested stage were randomly selected, each sample was comprised of five healthy plants, and nine samples were collected from three stages. After shaking off the loosely root-attached soil, the tightly adhered rhizosphere was collected with a sterile brush. The rhizosphere of a sample mixed together, and stored at −70 °C for microbial analysis. Seedling measurements were based on these five healthy plants, pooled into a single sample.

### DNA extraction and PCR amplification

Total genome DNA was extracted using cetyltrimethylammonium bromide method (Toju et al., 2013) and monitored on 1% agarose gel. The V4 region of the 16S rRNA gene was amplified with the primer pair 515F/806R, and the V4 region of the 18S rRNA gene with the primer pair 528F/706R. All PCR reactions were carried out in 30 µL reaction solutions with 15 µL of Phusion^®^ High-Fidelity PCR Master Mix (New England Biolabs, Ipswich, MA, USA), 0.2 µm of the forward and reverse primers, and about 10 ng template DNA. The PCR process included 98 °C for 1 min, 30 cycles of 98 °C for 10 s, 50 °C for 30 s and 72 °C for 30 s, finally 72 °C for 5 min.

### Illumina sequencing

The amplicons were mixed in equimolar amounts and submitted for sequencing to the IlluminaHiSeq (PE250; San Diego, CA, USA) platform at Novogene Science and Technology Co., Ltd. (Beijing, China). Clean tags were obtained by cutting off the barcodes and primer sequences, merging reads using FLASH (Magoč & Salzberg, 2011) to produce raw tags, and filtering the raw tags (Bokulich et al., 2013). Eventually, effective tags were gained by chimera removal (Edgar et al., 2011; Haas et al., 2011).

### OTU cluster and species annotation

All the effective tags were analyzed using Uparse (Version 7.0.1001) and clustered by their operational taxonomic units (OTUs) according to ≥97% sequence identity (Edgar, 2013). For each representative sequence, the Silva Database (Version 123) was used to annotate taxonomic information for bacteria and fungi by the Mothur and RDP classifier (Version 2.2) respectively (Wang et al., 2007; Quast et al., 2013). Multiple sequence alignment was conducted by MUSCLE (Version 3.8.31) (Edgar, 2004). OTU abundance was normalized with the least sequences according to the sample sequence number by Novogene Cloud Platform (www.novogene.com). Venn diagrams were generated using custom R scripts.

### Microbial diversity and functional prediction

All indices of alpha-diversity were calculated with QIIME (Version 1.7.0) and displayed with R (Version 2.15.3), comprising the abundance-based coverage estimation for bacteria (ACEB) and fungi (ACEF), and Shannon index of bacteria (SHAB) and fungi (SHAF). Alpha-diversities were analyzed by one-way ANOVA and multiple comparisons with Tukey test in SPSS (Version 22.0). Beta-diversity was calculated under Bray–Curtis by QIIME (Version 1.7.0). The functional prediction of microbes was based on the FAPROTAX database for bacteria (Louca, Parfrey & Doebeli, 2016) and the FUNGuild database for fungi (Nguyen et al., 2016). Principal coordinate analysis was displayed using the vegan, plyr, and ggplot2 packages and tested by Adonis based on Bray–Curtis in R (Version 2.15.3). Correlation between pathogens and plant growth was calculated by Pearson in SPSS (Version 22.0).

### Core microbe definition, significant-changing microbes and specific microbes

The core microbes for *A. sinensis* seedlings comprise OTUs which should be present in all nine samples, and each OTU contains at least three reads in every sample. By Novogene Cloud Platform, core OTUs were screened, and core OTU abundance was normalized with the most sequences according to the sample sequence number. Significant-changing microbes between growth stages were measured by *T*-test in SPSS (Version 22.0). Microbes that only dwelt on the certain growth stage were found by MS Excel 2010 and were considered as the stage-specific microbes. Microbes that were positively and significantly related to plant growth indices including plant weight, root length and diameter were identified by Spearman correlations (SPSS version 22.0) and were considered as the host-specific microbes.

### Environmental factors

Plant weight (PW), root length (RL), root diameter (RD), soil pH (pH), rainfall (RF), and climate temperature (T) were considered as the environmental factors in this study. PW was measured as the weight of the seeding without the root soil, RL as the length along the main root, and RD as the diameter 1.0 cm below the rhizome of the seedling. pH was determined with a glass electrode in water-to-soil ratio of 3:1 (v/w). RF and T data were collected from a website (http://data.cma.cn/). Environmental factors were tested by one-way ANOVA and multiple comparisons with Tukey test in SPSS (Version 22.0). Canonical correspondence analysis and Variation partitioning analysis were performed using the vegan package in R (Version 2.15.3).

## Results

### Global microbial composition

**Figure 1:**
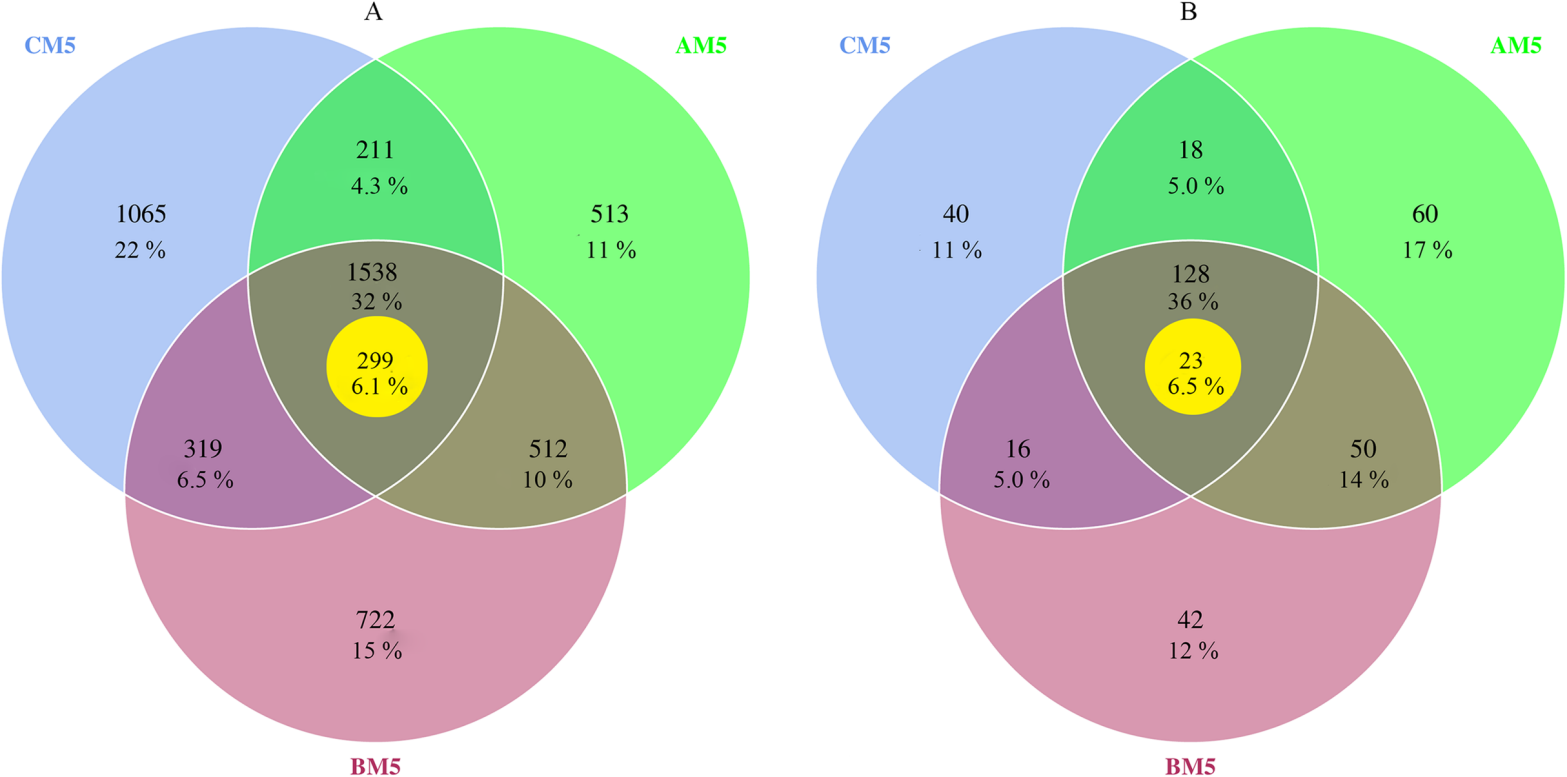
Venn diagrams of bacterial (A) and fungal (B) OTUs between different growth stages. % Indicates the number of OTUs in percentage of total OTUs. The yellow circles indicate core microbes. Overlap areas represent the shared OTUs. Bacteria: 1,538 overlap OTUs in all three stages; 512 overlap OTUs between AM5 and BM5, 319 overlap OTUs between BM5 and CM5, and 211 overlap OTUs between CM5 and AM5; and 513 OTUs in AM5, 722 OTUs in BM5, and 1,065 OTUs in CM5. Fungi: 128 overlap OTUs in all three stages; 50 overlap OTUs between AM5 and BM5, 16 overlap OTUs between BM5 and CM5, and 18 overlap OTUs between CM5 and AM5; and 60 OTUs in AM5, 42 OTUs in BM5, and 40 OTUs in CM5.

The high-quality reads, 559,472 for bacteria and 589,618 for fungi, were obtained from nine samples, and 198,477 bacterial reads and 34,848 fungal reads were used in the study after normalizing with the least sequences. The 4,880 bacterial and 354 fungal OTUs were present during growth stages. However, only 1,538 bacterial and 128 fungal OTUs were observed during all three stages, and other OTUs were distributed in three growth stages, respectively (Figs. 1A and 1B). Global microbes were classified into different levels (Table 1).

**Table 1:** Taxonomic levels of global and core microbes. % Indicates the number of core bacteria/fungi in percentage of global bacteria/fungi on taxonomic levels.

Microbe	Phylum	Class	Order	Family	Genus	Species
Global bacteria	30	61	127	233	553	274
Global fungi	8	31	69	98	122	104
Core bacteria	9 (30%)	19 (31%)	40 (31%)	54 (23%)	100 (18%)	35 (13%)
Core fungi	4 (50%)	11 (35%)	15 (22%)	16 (16%)	15 (12%)	9 (9%)

**Figure 2:**
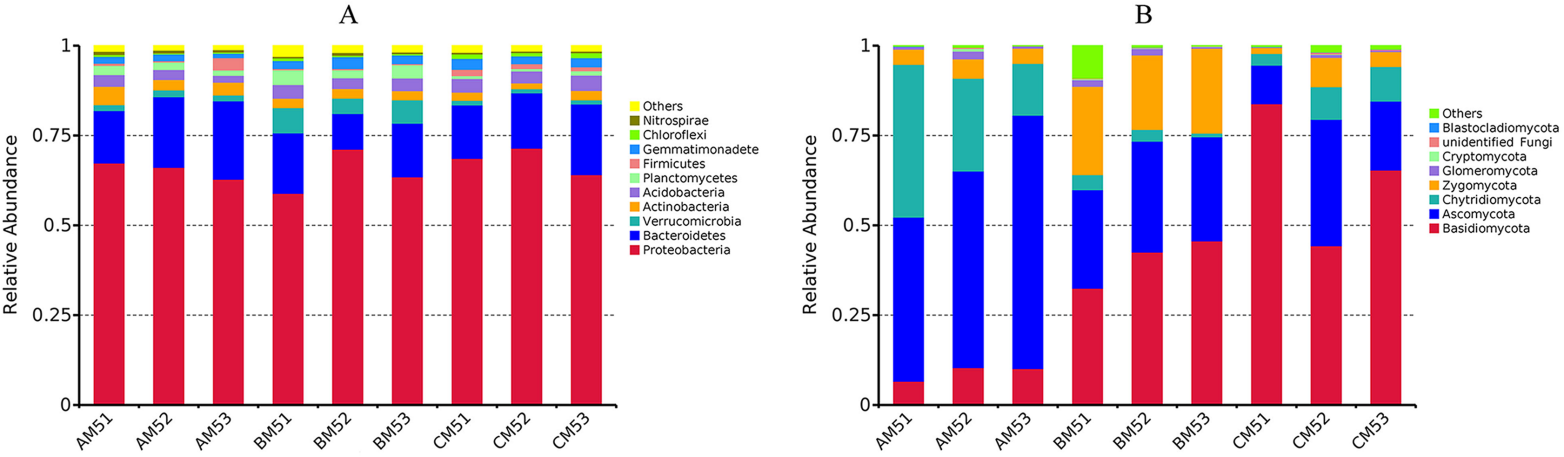
Relative abundances of the top ten phyla in bacteria (A) and the top eight phyla in fungi (B) at each growth stage. Others: the phyla without the top ten phyla in bacteria (A) and without the top eight phyla in fungi (B). AM51, AM52, and AM53, three parallels for AM5; BM51, BM52, and BM53, three parallels for BM5; and CM51, CM52, and CM53, three parallels for CM5.

The composition of bacteria and fungi was analyzed at the phylum and class levels. Proteobacteria and Bacteroidetes were the dominant phyla (relative abundance >10%) at each growth stage, Proteobacteria accounting for 59–72% and Bacteroidetes 10–22% (Fig. 2A). At the class level, Beta-, Alpha- and Gammaproteobacteria were the top three classes, and the sum of relative abundance was more than 50%. Fungal dominant phyla varied with growth stages. Ascomycota and Chytridiomycota were the dominant phyla (relative abundance >10%) in AM5, Basidiomycota, Ascomycota and Zygomycota in BM5, and Basidiomycota and Ascomycota in CM5. Ascomycota, Basidiomycota, Chytridiomycota, and Zygomycota accounted for 11–71%, 33–84%, 14–42%, and 21–25%, respectively (Fig. 2B).

### Microbial diversity and potential microbial functions

**Figure 3:**
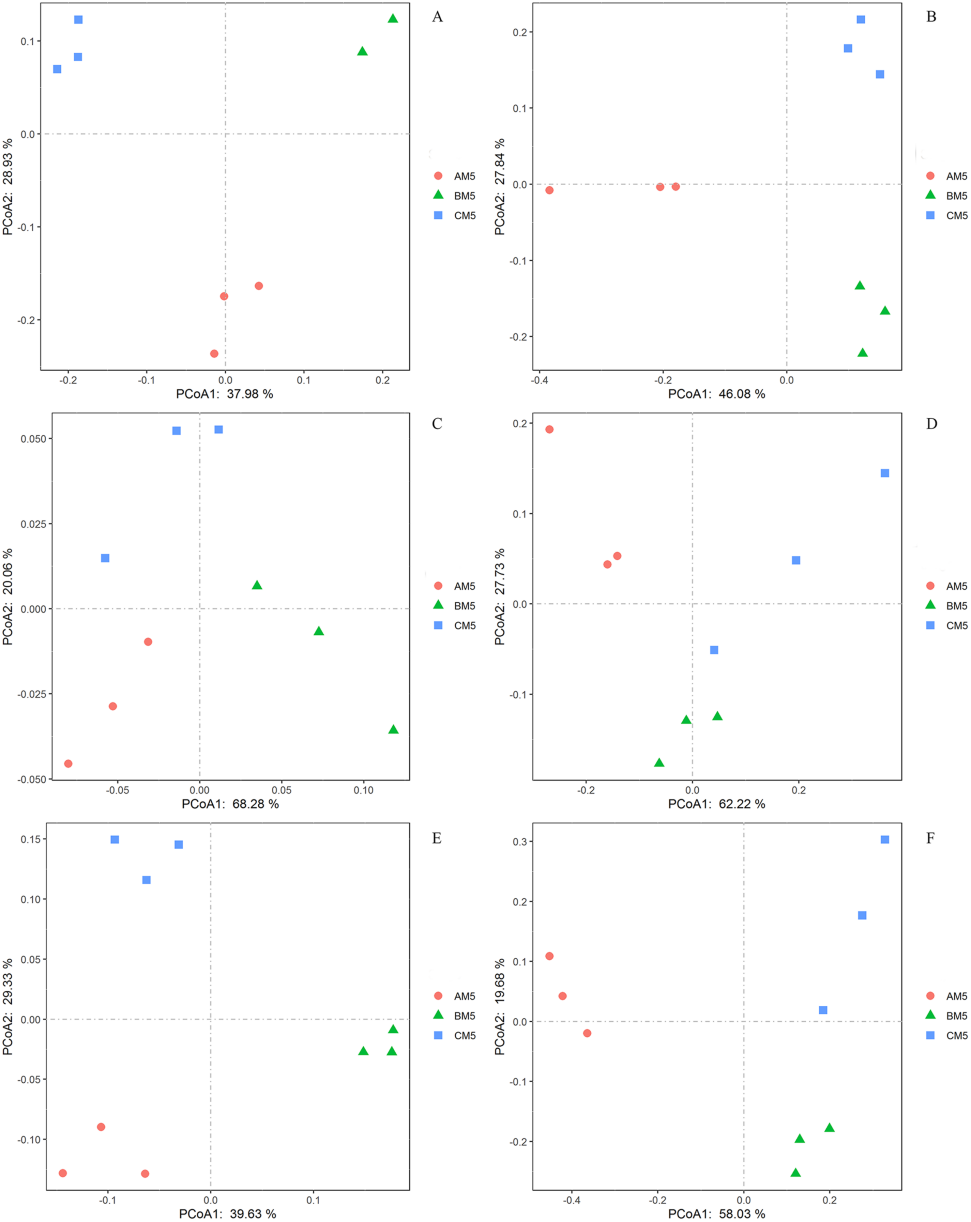
Principal coordinate analyses in the compositions of the global bacteria (A) and fungi (B) communities, the functions of the global bacteria (C) and fungi (D) communities, and the compositions of the core bacteria (E) and fungi (F) communities.

We analyzed the alpha- and beta-diversities of the microbial community. There was no significant difference in the alpha-diversity of the bacteria and fungi (Table 2), revealing that the community diversity was stable from AM5 to CM5. Principal coordinate analysis showed that the community compositions (beta-diversity) in the bacteria and fungi significantly (*P* < 0.01) changed with the growth stages (Figs. 3A and 3B).

**Table 2:** Alpha-diversity of microbial communities. Data are presented as mean ± standard error (SE), *n* = 3. Different lowercase letters indicate statistically significant (*P* < 0.05) differences. ACEB, abundance-based coverage estimation for bacteria; SHAB, Shannon index of bacteria; ACEF, abundance-based coverage estimation for fungi; and SHAF, Shannon index of fungi.

Stage	Bacterial diversity		Fungal diversity	
	ACEB	SHAB	ACEF	SHAF
AM5	2578.94 ± 100^a^	8.10 ± 0.13^a^	160.91 ± 21^a^	4.58 ± 0.26^a^
BM5	3140.67 ± 321^a^	8.58 ± 0.04^a^	155.77 ± 21^a^	4.78 ± 0.24^a^
CM5	3215.68 ± 63^a^	8.37 ± 0.14^a^	134.00 ± 15^a^	3.53 ± 0.69^a^

**Figure 4:**
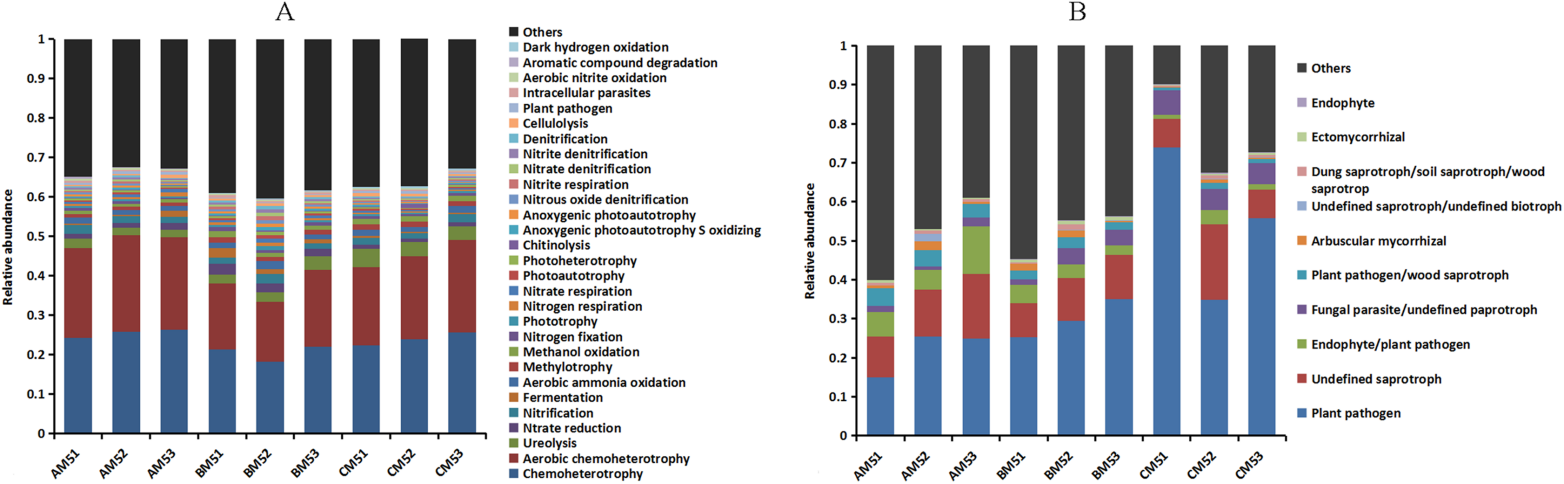
Relative abundances of the bacterial (A) and fungal (B) community functions at each growth stage. Others: the functions without the top thirty functions in bacteria and without the top ten functions in fungi.

The functions of the bacteria included the biotransformation of C, N, S and Fe, pollutant degradation, and plant pathogens (Fig. 4A). The functional groups of the fungi community included plant pathogens, saprotrophs, and mycorrhizae (Fig. 4B). Principal coordinate analysis showed that the functions of the bacterial (Fig. 3C) and fungal (Fig. 3D) communities changed obviously (*P* < 0.01) with seedling growth. Among these functions, the relative abundance of fungal pathogens increased with plant growth, and significantly (*P* < 0.05) correlated with PW, RL and RD (Table 3).

**Table 3:** Correlation between plant growth indices, genera and fungal pathogens. *Itersonilia* and *Dioszegia* are the fungal genera, and other genera are the bacterial genera. PW, plant weight; RL, root length; and RD, root diameter.

Genera	Method	PW	RL	RD
*Massilia*	Spearman	0.93**	0.88**	0.92**
*Ramlibacter*	Spearman	0.90**	0.77*	0.85**
*Methylotenera*	Spearman	0.73*	0.80**	0.82**
*Anaeromyxobacter*	Spearman	0.94**	0.88**	0.96**
*Lysobacter*	Spearman	0.90**	0.73*	0.85**
*Itersonilia*	Spearman	0.92**	0.91**	0.90**
*Dioszegia*	Spearman	0.90**	0.83**	0.87**
Fungal pathogens	Pearson	0.78*	0.73*	0.75*

**Note:**

* and ** represent significance at *P* < 0.05 and 0.01, respectively.

### Core microbes, significant-changing microbes and specific microbes

We found the core bacteria and fungi OTUs. The 299 bacterial OTUs (158,940 reads) (Fig. 1A) were defined as the core bacteria, being 6.1% of the total bacterial OTUs. The 23 fungal OTUs (17,541 reads) (Fig. 1B) were defined as the core fungi, accounting for 6.5% of the total fungal OTUs. Principal coordinate analysis showed that the bacterial and fungal core communities significantly (*P* < 0.01) changed in their composition throughout the growth stages (Figs. 3E and 3F). The core microbes were classified into different levels (Table 1).

The bacterial and fungal genera that significantly (*P* < 0.05) changed between the growth stages were found (Table 4), and among them, 14 bacterial and four fungal genera obviously (*P* < 0.05) changed throughout the growth stages (Table 4). Bacteria and fungi only dwelling on the certain growth stage were found (Table 5). In the dominant phyla, five bacterial and two fungal genera were significantly and positively correlated with PW, RL and RD (Table 3).

**Table 4:** Significant-changing genera between AM5 and BM5 and between BM5 and CM5. Thirty-five bacterial and eight fungal genera changed significantly (*P* < 0.05) between AM5 and BM5, and 64 bacterial and six fungal genera between BM5 and CM5. The underlined genera obviously (*P* < 0.05) changed throughout the growth stages.

Genera	Between AM5 and BM5	Between BM5 and CM5
Bacteria	*Sphingomonas*, *Massilia*, *Arenimonas*, *Reyranella*, *Fluviicola*, *Panacagrimonas*, *Haloferula*, *Verrucomicrobium*, *Rickettsia*, *Hydrotalea*, *Candidatus Methylacidiphilum*, unidentified MWH-CFBk5, unidentified BSV26, *Pedobacter*, *Sphingobium*, *Flavisolibacter*, *Gemmatimonas*, *Adhaeribacter*, *Hymenobacter*, *Sorangium*, *Bacillus*, Pir4 *lineage*, *Xanthomonas*, *Roseomonas*, *Spirosoma*, *Turneriella*, *Illumatobacter*, *Perlucidibaca*, *Byssovorax*, *Euzebya*, *Nibribacter*, *Skermanella*, *Kribbella*, *Paracocccus*, *Bythopirellula*	*Sphingomonas*, *Massilia*, *Arenimonas*, *Reyranella*, *Fluviicola*, *Panacagrimonas*, *Haloferula*, *Verrucomicrobium*, *Rickettsia*, *Hydrotalea*, *Candidatus Methylacidiphilum*, unidentified MWH CFBk5, unidentified BSV26, *Rhizobium*, *Anaeromyxobacter*, *Devosia*, *Blastocatella*, *Bryobacter*, *Hydrogenophaga*, *Mesorhizobium*, *Candidatus Solibacter*, *Sphingopyxis*, *Pirellula*, *Candidatus Koribacter*, *Anaerolinea*, *Chryseobacterium*, *Brevundimonas*, *Polycyclovorans*, *Phaselicystis*, *Chryseolinea*, *Chitinophaga*, *Schlegelella*, *Aeromicrobium*, *Lysinimonas*, *Deferrisoma*, *Solirubrobacter*, *Tumebacillus*, *Geobacter*, *Pseudonocardia*, unidentified *Gemmatimonadetes*, CL500-29 marine group, *Intestinibacter*, *Sporichthya*, unidentified *Gaiellales*, unidentified *Anaerolineaceae*, unidentified *Planctomycetaceae*, *Intrasporangium*, *Nitrosomonas*, *Acidothermus*, *Thermincola*, *Clostridium* sensu stricto 1, *Effusibacillus*, *Geothermobacter*, *Terrabacter*, *Syntrophobacter*, *Paucimonas*, *Isosphaera*, *Kineococcus*, *Rhodomicrobium*, *Magnetospirillum*, unidentified DB1-14, *Christensenellaceae* R-7 group, *Clostridium* sensu stricto 8, *Frankia*
Fungi	unidentified *Endogonales*, *Cladosporium*, unidentified *Agaricomycetes*, *Phoma*, *Itersonilia*, *Cochliobolus*, unidentified *Entylomatales*, *Scopulariopsis*	unidentified *Endogonales*, *Cladosporium*, *Phoma*, unidentified *Agaricomycetes*, unidentified *Xylariales*, *Sporobolomyces*

**Table 5:** Bacteria and fungi dwelling on the certain growth stage. Nine bacterial and 21 fungal genera dwelt on AM5, six bacterial and seven fungal genera on BM5, and 55 bacterial and four fungal genera on CM5.

		Genus
AM5	Bacteria	*Lactobacillus*, *Promicromonospora*, *Pirellula*, unidentified GR-WP33-30, *Amycolatopsis*, *Coxiella*, *Rhizocola*, Pir4 lineage, *Aquicella*
	Fungi	*Volvariella*, *Emericellopsis*, *Tilletiaria*, *Metarhizium*, *Ochroconis*, *Acremonium*, *Paraglomus*, *Rhizoctonia*, *Naohidea*, *Octosporella*, *Bionectria*, *Helvella*, *Thecotheus*, *Endogone*, *Arthrobotrys*, *Acaulospora*, *Paratritirachium*, *Syncephalis*, *Leptosphaeria, Hyphodontia*, *Neobulgaria*
BM5	Bacteria	unidentified MWH-CFBk5, *Asteroleplasma*, *Flavobacterium*, *Candidatus* *Methylacidiphilum*, *Ferruginibacter*, *Prosthecobacter*
	Fungi	*Melampsora*, *Sistotrema*, *Gymnoascus*, *Hannaella*, unidentified* Plectosphaerellaceae*, *Nowakowskiella*, *Synchytrium*
CM5	Bacteria	*Haliangium*, *Simiduia*, *Roseiflexus*, *Phaselicystis*, *Geothermobacter*, *Leptolyngbya*, *Lutispora*, *Thermincola*, *Haliscomenobacter*, *Ferrovibrio*, *Anaerolinea*, *Parvularcula*, *Candidatus* *Koribacter*, *Hyphomonas*, unidentified 34P16, unidentified Clostridiaceae 1, *Isosphaera*, *Effusibacillus*, Clostridium sensu stricto 12, *Desulfurispora*, *Desulfovirga*, *Syntrophus*, *Bdellovibrio, Intestinibacter*, *Acidothermus*, *Rhodomicrobium*, *Gaiella*, *Flavisolibacter*, *Azospirillum*, *Defluviicoccus*, unidentified *Gaiellales*, *Inhella, Denitratisoma*, *Microvirga*, Clostridium sensu stricto 8, *Oxalophagus*, *Tumebacillus*, *Syntrophobacter*, Christensenellaceae R-7 group, *Herpetosiphon*, *Micromonospora*, Clostridium sensu stricto 5, *Catenuloplanes*, *Desulfosporosinus*, *Leadbetterella*, *Sandaracinobacter*, unidentified mle1-27, Ruminiclostridium 1, *Fonticella*, *Anaeromyxobacter*, Clostridium sensu stricto 10, *Singulisphaera*, *Deferrisoma*, unidentified *Gemmatimonadetes*, *Acidibacter*
	Fungi	*Cochlonema*, *Mrakia*, *Atractiella*, unidentified* Capnodiales*

### Environmental factors

**Figure 5:**
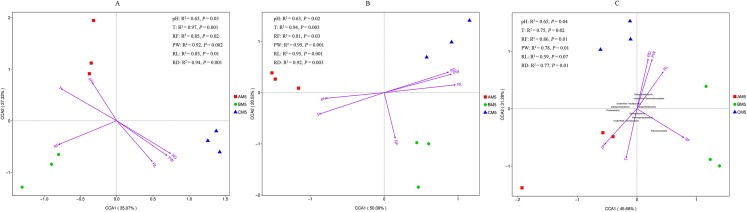
Canonical correspondence analysis between environmental factors and bacterial OTUs (A), between environmental factors and fugal OTUs (B), and between environmental factors and bacterial classes (C). The top 10 bacterial classes are displayed in C. PW, plant weight; RL, root length; RD, root diameter; pH, soil pH; RF rainfall; and T, climate temperature.

Except for pH, other environmental factors significantly (*P* < 0.01) changed during the growth stages (Table 6). Canonical correspondence analysis showed that pH, T, RF, RD, PW and RL were the key divers to microbial community composition (Figs. 5A and 5B). AM5 had positive correlation with pH and T, indicating that they were the major drivers shaping community composition to AM5. Same as above analysis, RF was the major driver to BM5, and RD, RW and RL were the major drivers to CM5. Additionally, alpha- and gammaproteobacteria were sensitive to RF and T respectively. Beta- and Deltaproteobacteria were closely correlated to the plant growth, and they could adapt to the low temperature environment (Fig. 5C).

**Table 6:** Environmental factor variation with growth stages. Data are presented as mean ± SE, *n* = 3. Different lowercase letters indicate statistically significant (*P* < 0.05) differences. PW, plant weight; RL, root length; RD, root diameter; pH, soil pH; RF, rainfall; and T, climate temperature.

Stage	PW (g)	RL (cm)	RD (cm)	pH	T (°C)	RF (mm/d)
AM5	0.39 ± 0.09^c^	6.00 ± 0.17^b^	1.58 ± 0.11^c^	8.33 ± 0.09^a^	17.33 ± 1.03^a^	2.64 ± 0.04^b^
BM5	2.83 ± 0.57^b^	10.43 ± 0.71^a^	4.47 ± 0.61^b^	8.00 ± 0.03^a^	14.43 ± 2.00^ab^	4.47 ± 0.27^a^
CM5	5.85 ± 0.64^a^	12.19 ± 0.87^a^	8.68 ± 0.48^a^	7.91 ± 0.18^a^	9.55 ± 1.10^ab^	2.11 ± 0.04^b^

**Figure 6:**
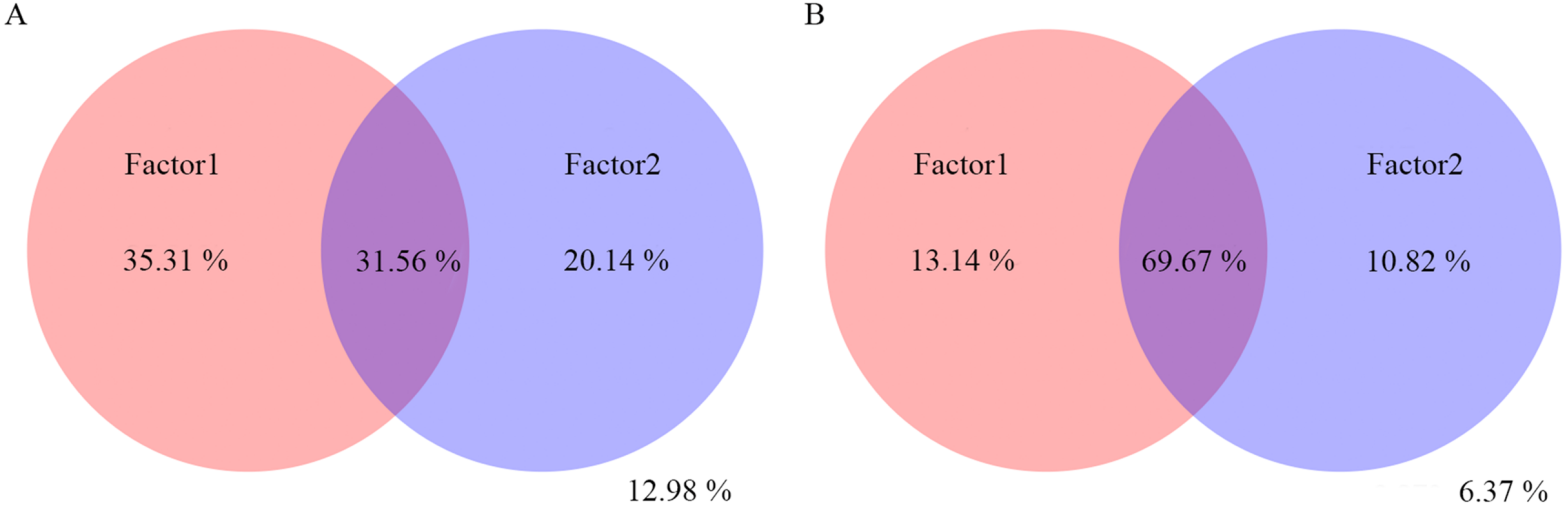
Variation partitioning analysis of the effects of pH, T, RF, RD, PW and RL on the bacterial (A) and fungal (B) community composition. The variance of 35.31%, 20.14% and 31.56% for bacterial community could be explained by factor1, factor2, and both factor1 and factor2, respectively. The variance of 12.98% could not be explained by factor1 and factor2. The variance of 13.14%, 10.82% and 69.67% for fungal community could be explained by factor1, factor2, and both factor1 and factor2, respectively. The variance of 6.37% could not be explained by factor1 and factor2.

Variation partitioning analysis showed that most of the variation in bacterial and fungal communities can be explained by environmental factors (Figs. 6A and 6B). Factors1 (pH, T and RF) contributed more variation to microbial community composition than factors2 (RD, PW and RL).

## Discussion

In our study, Proteobacteria and Bacteroidetes were the dominant phyla. They also were the dominant phyla in other plant rhizosphere. Compared to the previous works, the Proteobacteria relative abundance in this study was higher than that in these plants, including ramie (Zhu et al., 2018), tomatoes (Shao et al., 2018), potatoes (Weinert et al., 2011), and *Arabidopsis* (Bulgarelli et al., 2012). This implies that Proteobacteria is generally adapted to the rhizosphere environment across diverse plant species. In terms of fungi, there were the distinct dominant phyla between the growth stages, but these dominant phyla, Basidiomycota, Ascomycota, Chytridiomycota and Zygomycota, have been identified as dominant phyla in previous studies on *Panax notoginseng* (Tan et al., 2017), ramie (Zhu et al., 2018), and wheat and canola (Schlatter et al., 2019). However, unlike plant and animal ecology, there is not a clear definition for the dominant phylum in microbial ecology until now.

Previous studies have shown that the alpha-diversity of the rhizosphere bacterial and fungal communities does not significantly change with plant development, but the beta-diversity significantly changed (Chaparro, Badri & Vivanco, 2014; De Souza et al., 2016; Zimudzi et al., 2018). The similar results were also found in this study. Thus we speculate that the abundance of some microbes could significantly change between growth stages, or the microbes that dwelt on the certain stage could be present in microbial community succession.

Notably, the beta-diversity and function of the bacterial and fungal communities significantly changed with the growth stages, suggesting that microbial community composition variation was accompanied by the changes in the community function (Philippot, Raaijmakers & Van Der Putten, 2013). Some plant pathogens were present during the growth stages, such as *Pseudomonas viridiflava* (Albu et al., 2018), *Rhodococcus fascians* (Putnam & Miller, 2007), *Rhizobium larrymoorei* (Bouzar & Jones, 2001), and *Rhizoctonia solani* (Fang, 1983). *R. solani* that involved in *A. sinensis* root rot as one of the pathogens was present in this study, indicating that *R. solani* was an opportunistic pathogen. Normally, *R. solani* may have a neutral relationship with the host plant, but if the plant is stressed, then this relationship can change to cause the plant disease.

The core microbes were a subset (less than 7.0%) of global microbiome, which could facilitate the design of plant growth promoting rhizobacteria for *A. sinensis* seedlings. However, we find that the percentage of core microbes in different studies varies widely (De Souza et al., 2016; Pfeiffer et al., 2017), and the fact that the core microbial community composition significantly changed during the growth stage in our study is contrary to the previous findings (De Souza et al., 2016; Pfeiffer et al., 2017). These may be mainly caused by the distinct definition of core microbes in different studies (Lundberg et al., 2012; Saunders et al., 2016; Meier, Avis & Phillips, 2013). Among the significant-changing bacterial genera, 43% of them belonged to Proteobacteria phylum, for example, the *Sphingomonas*, *Rickettsia* and *Reyranella* of Alphaproteobacteria, the *Massilia* of Betaproteobacteria, and the *Arenimonas* and *Panacagrimonas* of Gammaproteobacteria.

The stage-specific microbes could be used as the indicators of a growth stage. Those on the rhizosphere are in low abundance and could be more susceptible to ecological drift (Lankau, Hong & Mackie, 2012). It has been shown that low abundance microbes can play an important ecological function, for example, in host health (Nuccio et al., 2016) and microbial community stability and diversity (Shade et al., 2014). Root exudates as a nutrient play an important role for rhizosphere microbial recruitment (Reinhold-Hurek et al., 2015). So we surmise that the stage-specific microbes could be related to the special root exudates inducing the fast response of microbes (Zhang, Vivanco & Shen, 2017).

Five bacterial and two fungal genera were considered as the host-specific microbes. For bacteria, *Massilia* is a major group of bacteria associated with many plants. Members of *Massilia* were reported to show plant growth promotion traits, including indole acetic acid production (Kuffner et al., 2010), siderophore production (Chimwamurombe et al., 2016), and biocontrol against plant-pathogenic fungi (Adrangi et al., 2010). Other bacteria are also reported with plant growth promotion, such as *Methylotenera* related to C cycling (Kalyuzhnaya et al., 2010), *Ramlibacter* to N and P cycling (Props et al., 2019), *Lysobacter* to the suppression of soil phytopathogens (Lazazzara et al., 2017), *Anaeromyxobacter* to Fe reduction (Suriyavirun et al., 2019), and *Devosia* to root nodules and nitrogen fixation (Rivas et al., 2002). For fungi, *Itersonilia* is known as a fungal pathogen. For example, *Itersonilia perplexans* appearing in this study causes petal, foliar, and seedling blight and root cankers on host plants (McGovern et al., 2006). Overall, the beneficial microbes and plant pathogens dwelt on a balanced microbial ecosystem of *A. sinensis* seedlings (Vimal et al., 2017).

In our results, pH, T, RF, RD, PW and RL were the important drivers for the bacterial and fungal community composition, which were consistent with previous studies (Chaparro, Badri & Vivanco, 2014; Liang et al., 2015; Aslam et al., 2016; Nuccio et al., 2016).

Previous reports have described the similar results that Alpha-, Beta-, and Gammaproteobacteria were the predominant classes in the plant rhizosphere (Cardinale et al., 2015; Gottel et al., 2011). According to the sensitivity of the alpha-, beta-, delta-, and gammaproteobacteria to environmental factors, the plant growth promoting rhizobacteria for *A. sinensis seedlings* should be from the members of Beta- and Deltaproteobacteria. In short, Proteobacteria variation in our study confirmed that Proteobacteria are r-strategists, able to quickly adapt to a changing environment (Brzeszcz et al., 2016).

Finally, all of results in our study are based on the taxonomic relative abundance, but the reported studies have demonstrated that the observed differences with relative abundances can cover those with the actual taxonomic abundances (Zhang et al., 2017; Tkacz, Hortala & Poole, 2018). The method of combining the quantification and relative abundance of the microbial communities should be used in the further studies (Lou et al., 2018; Guo et al., 2019).

## Conclusions

The study for the first time reported the variation and environmental drivers of rhizosphere bacterial and fungal communities during the growth of *A. sinensis* seedlings. Bacterial dominant phyla were Proteobacteria and Bacteroidetes, and fungal dominant phyla were Ascomycota, Basidiomycota, Chytridiomycota and Zygomycota. The variation in microbial community composition was accompanied by community function changes. We identified the core microbes, significant-changing microbes, stage-specific microbes, and host-specific microbes. Fungal pathogen relative abundance increased with plant growth. *R. solani* was an opportunistic pathogen that involved in *A. sinensis* root rot. Therefore, the study increased the understanding of the rhizosphere bacterial and fungal communities of *A. sinensis* seedlings. In further studies, the relationship between root exudates and stage-specific microbes should be investigated. In addition, a method with the combination of quantitative and relative abundance of microbial communities could contribute to a better understanding for population variation.

## Additional Information and Declarations

### Data Availability

The following information was supplied regarding data availability: The sequence is available at the NCBI Short Read Archive: SRP153248.
